# Pressure Differences from Clear Aligner Movements Assessed by Pressure Sensors

**DOI:** 10.1155/2020/8376395

**Published:** 2020-03-12

**Authors:** Ho-jung Son, Kyeong-ho Lee, Ji-young Sim, Hae-young Kim, Ji-hwan Kim, Woong-chul Kim

**Affiliations:** ^1^Department of Dental Laboratory Science and Engineering, College of Health Science, Korea University, Seoul, Republic of Korea; ^2^School of Electrical Engineering, College of Engineering, Korea University, Seoul, Republic of Korea; ^3^Department of Public Health Sciences, Graduate School, Korea University, Seoul, Republic of Korea

## Abstract

**Objectives:**

In this study, a clear aligner was moved at intervals of 0.25 mm and pressure variations were assessed using a sensor.

**Methods:**

The model used for producing the clear aligner was created using a 3-dimensional printer. A clear, circular thermoplastic of 0.75 mm thickness was used for making the clear aligner using the vacuum forming method. A pressure sensor was used to assess the pressure in the device, and the variation in the sheet pressure was statistically analyzed tooth movement using the clear aligner, moving at an interval of 0.25 mm, is recommended.

**Results:**

The results of pressure sensor assessment showed that the pressure of the devices with 0.25 mm and 1.00 mm movements was identical to that of the device with 0 mm movement. In other words, the pressure sensor could not distinguish the pressure of devices that moved 0.25 mm and 1.00 mm.

**Conclusions:**

This experiment demonstrated that a movement of more than 0.50 mm is needed to apply the appropriate pressures needed for orthodontics in a clear polymer sheet.

## 1. Introduction

**Figure 1:**
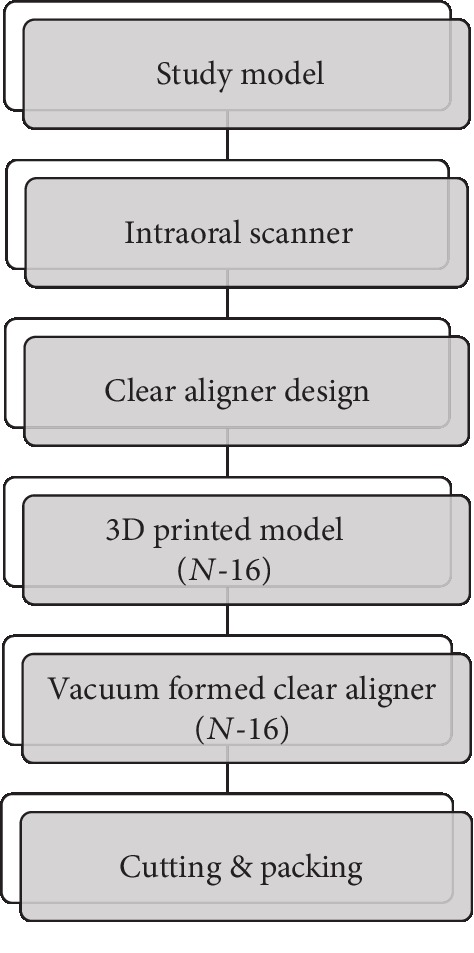
Sequence of producing a clear aligner.

Orthodontic treatment is performed for functional and aesthetic satisfaction in cases of dentofacial malocclusion. The number of patients who received orthodontic treatments rose linearly in the late 20^th^ century [1]. Despite the necessity of orthodontics, adults tend to show negative attitudes toward wearing traditional orthodontic appliances, such as a wire, band, and bracket. Compared to conventional, fixed appliances, it has been reported that the clear aligner is more comfortable to wear and is less resorbed [2, 3]. Aesthetically agreeable solutions could be provided using computer assistance technology, which produces polymer-based clear materials to treat adult patients who need orthodontic treatment, through a clear orthodontic system [4–6]. The sequence of the clear orthodontic system is illustrated in Figure 1. Orthodontic treatment offers psychological treatment and improves the quality of periodontal and aesthetic treatment outcomes.

In 1945, Kesling demonstrated that consecutive tooth movement is possible by utilizing positioners and producing setup models in multiple phases [7]. Computer-aided designing (CAD)/computer-aided manufacturing was used to produce a model according to tooth movements in alignment technology, and thereafter, the Invisalign System (Align Technology, Santa Clara, California, USA) was used for making the device [8]. This progressed towards the usage of a clear alignment device, namely, the clear aligner. Previous studies on clear aligners relied on clinical cases of orthodontic treatment. Few studies on clear device materials or orthodontic force have been conducted so far due to the difficulty in assessment of orthodontic force. However, developments in sensor technology have produced devices capable of assessing this force [9]. Along these lines, this study is aimed at assessing the orthodontic force of clear aligners. The clear aligner is produced in accordance with the tooth form after undergoing a series of plastic procedures. This orthodontic device is made after predicting the posterior phases of tooth movements. The ideal tooth movements are recommended to be from 0.25 mm to 0.33 mm [4]. If the amount of movement of the clear aligner is not appropriate, the intended tooth movement is not achieved. This study examined whether necessary orthodontic force can be obtained if the clear aligner is moved at an interval of 0.25 mm. An ideal orthodontic force minimizes tissue damage and generates the optimal force that maximizes the speed of tooth movement [10]. By using an ideal orthodontic force and proper movements of clear aligners, efficient alignment with reduced alignment period could be achieved in patients.

In this study, 40–80 clear aligners were produced per patient and a new device was altered and dressed up at intervals of two weeks. The orthodontic force applied ranged from five to 10 g per single tooth. (The optimal force for the tipping movement of a single tooth ranges from 50 g to 75 g [11].).

## 2. Materials and Methods

**Figure 2:**
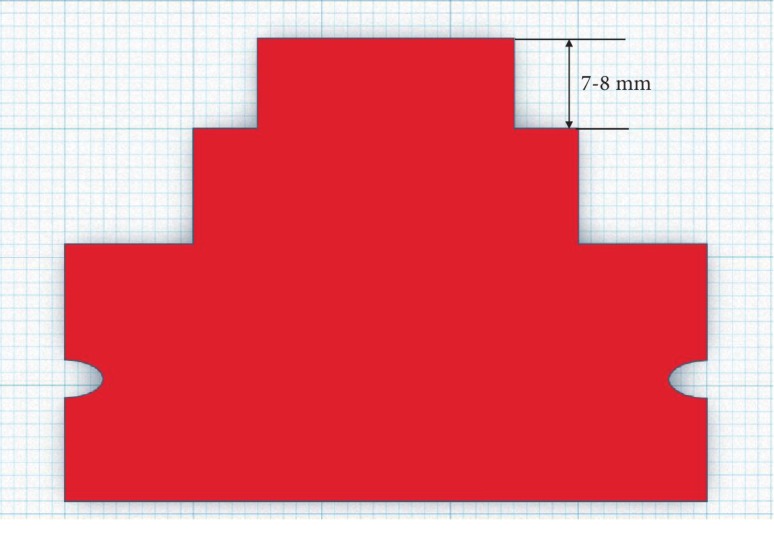
3D design to be produced by a clear alignment device.

**Figure 3:**
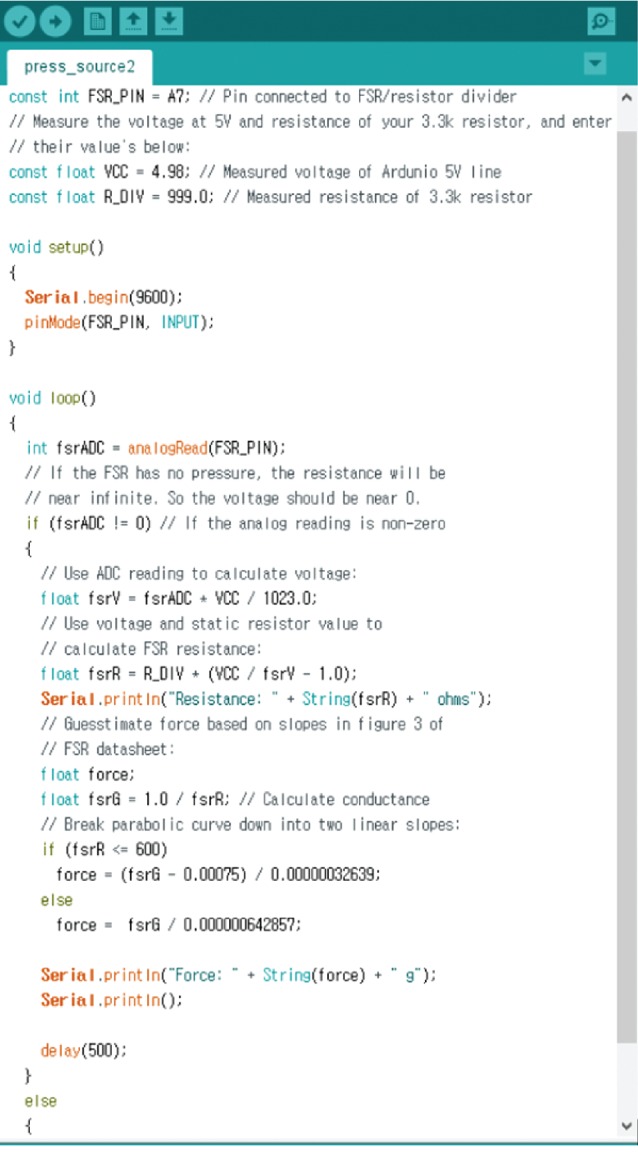
Arduino source for pressure sensors.

In this study, the mold to replace the teeth was designed using CAD. As illustrated in Figure 2, the distance between the central incision to the canine teeth was defined to be 8 mm. In addition, the relocation of the central incision from the canine teeth to the front was defined to be up to 7 mm. A model, relocating at an interval of 0.25 mm from 7 mm to 8 mm, was produced. It had to be designed in an angular form to assess the arch by utilizing a pressure sensor. A study model for producing the clear aligner was made by using a 3-dimensional printer. The designed model was placed on the vacuum pressing machine (Biostar, Scheu-Dental, Iserlohn, Germany) by using the currently available 0.75 mm thick polymer sheet (Duran, Scheu-Dental, Iserlohn, Germany), and the clear aligner for orthodontics was produced at 220°C and 5 bars. 16 polymer sheets were softened to produce models, and the samples were manufactured. A pressure sensor (FSR-402, Interlink Electronics, Santa Barbara, CA) was connected to a microprocessor (Arduino Nano, Arduino, Italy), and the codes were organized as shown in Figure 3.

**Figure 4:**
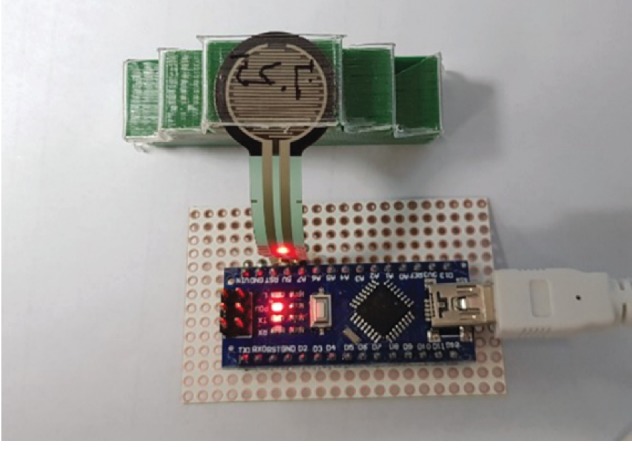
Measurement of tooth movement pressure using pressure sensor.

The orthodontic force was assessed at an interval of 0.25 mm on the synthesized model. As shown in Figure 4, clear aligners were assessed by installing them according to thickness. The thermoformed sheet was placed on the model with an 8 mm length between the central incision and the canine teeth. In this case, the gap between the model and the sheet is 0 mm. A 7.75 mm long sheet was placed on the 8 mm long model, and the orthodontic force was assessed by utilizing the pressure sensor that indicated 0.25 mm, which was the gap between the model and the sheet. This means that the force was identical with the 0.25 mm distance of movement. In addition, a 7.5 mm long sheet was placed on the 8 mm long model, and the orthodontic force was assessed by using the pressure sensor, which kept the force identical with the 0.5 mm distance of movement. Subsequently, a 7.5 mm long sheet was placed on the 8 mm long model, and the orthodontic force was assessed by using the pressure sensor, which kept the force identical with the 0.5 mm distance of movement. Next, a 7.25 mm long sheet was placed on the 8 mm long model, and the orthodontic force was assessed by using the pressure sensor. The gap between the model and the sheet was 0.75 mm, which kept the force identical with the 0.75 mm distance of movement. Finally, a 7 mm long sheet was placed on the 8 mm high model, and the orthodontic force was assessed by using the pressure sensor. The gap between the model and the sheet was 1.0 mm, which means that the force is equivalent with the 1.0 mm distance of movement. 16 samples were, respectively, assessed as described above. The pressure of the device was assessed by utilizing the pressure sensor. The variations of pressure in the sheet were statistically analyzed using SPSS.

## 3. Results

**Figure 5:**
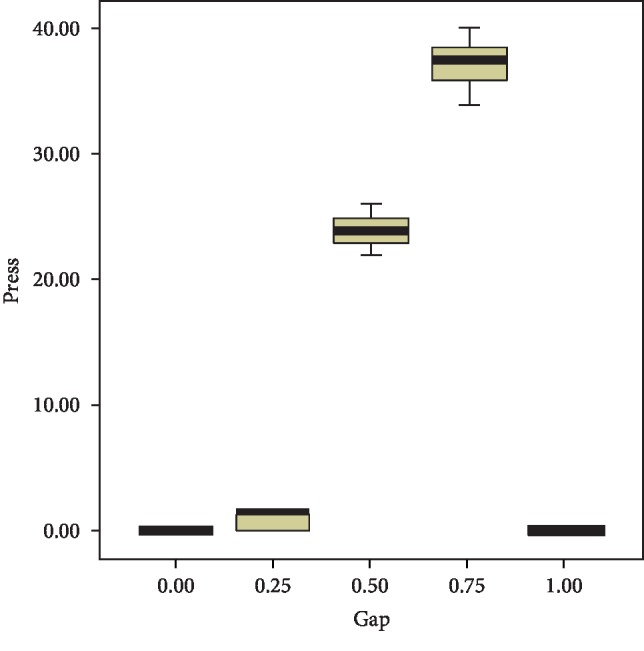
Graph of pressure due to the clear aligner and model gap.

The placement of the clear aligner shot above 8.0 mm on the model; at the height of 8.0 mm, it showed 0 g or 1.52 g orthodontic force. As shown in Table 1, the pressure value was identical with the 0 mm movement when a device with differences of 0.25 mm and 1.0 mm was installed. The measurement results are shown in Figure 5.

## 4. Discussion

Under constant force, the deflection of a viscoelastic material increases with time, and at constant deflection, its load decreases; therefore, creep and stress relaxation are the properties of clear aligners. The properties of the aligner material, time, and amount of activation affect the orthodontic force [12]. Excess force on the tooth causes pain, whereas negligible force provides no corrective effect [13, 14].

For curved contact surface between the model and the sensor, the measurement surface is made flat to prevent weight imbalance of the sensor [12]. The pressure sensor was not utilized unless the clear aligner and model made contact. The minimum value of force was 0 or 1.52 g when the clear aligner and the model made contact. The pressure sensor showed no readings between 0 and 1.52 g. The 0 mm, 0.25 mm, and 1.0 mm movements revealed minimum values of 0 g or 1.52 g. The average value was 1.05 g of weak force when moving 0.25 mm. However, the *p* value was found to be different from the case of not moving or having a gap of 1 mm. Moreover, force is not conveyed to the section to be actually assessed, since distortions first occurred in other sections if the clear aligner was moved by 1.00 mm. Therefore, it can be assumed that proper orthodontic force can be acquired by moving from 0.5 mm to 0.75 mm when clear aligners are manufactured.

## 5. Conclusion

The results of this study revealed that it is necessary to produce devices with at least more than 0.50 mm of movement range for orthodontic treatment using clear aligners, since the expected orthodontic force cannot be obtained if the movements are less than 0.50 mm. Furthermore, proper orthodontic force cannot be obtained at the preferred section since the clear aligner first makes contact with other parts if its movement exceeds 1.00 mm.

## Figures and Tables

**Table 1:** Significance, mean values, and standard deviations according to gap.

Group (mm)	*N*	*p* = .05			
		1	2	3	4
Gap 0.0	16	0.00 (0)	—	—	—
Gap 0.25	16	—	1.05 (0.73)	—	—
Gap 0.5	16	—	—	24.00 (1.10)	—
Gap 0.75	16	—	—	—	37.38 (1.82)
Gap 1.0	16	0.00 (0)	—	—	—
*p* value		1.00	0.001	0.000	0.000

Kruskal-Wallis test. Group variable: gap between the position of the model and the intersheet position.

## Data Availability

The [EXCEL TYPE] data used to support the findings of this study are available from the corresponding author upon request.

## References

[1] Bollen A. M., Cunha-Cruz J., Hujoel P. P. (2007). Secular trends in preadult orthodontic care in the United States: 1942-2002. *American Journal of Orthodontics and Dentofacial Orthopedics*.

[2] Miller K. B., McGorray S. P., Womack R. (2007). A comparison of treatment impacts between Invisalign aligner and fixed appliance therapy during the first week of treatment. *American Journal of Orthodontics and Dentofacial Orthopedics*.

[3] Miethke R. R., Brauner K. (2007). A comparison of the periodontal health of patients during treatment with the Invisalign® system and with fixed lingual appliances. *Journal of Orofacial Orthopedics*.

[4] Boyd R. L., Miller R., Vlaskalic V. (2000). The Invisalign system in adult orthodontics: mild crowding and space closure cases. *Journal of Clinical Orthodontics*.

[5] Sheridan J. (1993). Essix retainers: fabrication and supervision for permanent retention. *Journal of Clinical Orthodontics: JCO*.

[6] Lindauer S. J. (1998). Comparison of Essix and Hawley retainers. *Journal of Clinical Orthodontics*.

[7] Kesling H. D. (1945). The philosophy of the tooth positioning appliance. *American Journal of Orthodontics and Oral Surgery*.

[8] Vlaskalic V., Boyd R. (2001). Orthodontic treatment of a mildly crowded malocclusion using the Invisalign system. *Australian Orthodontic Journal*.

[9] McAuliffe P., Kim J. H., Diamond D., Lau K., O'connell B. (2015). A sleep bruxism detection system based on sensors in a splint-pilot clinical data. *Journal of Oral Rehabilitation*.

[10] Burstone C. J. (1989). The biophysics of bone remodeling during orthodontics (optimal force considerations). *The biology of tooth movement*.

[11] Proffit W. R., Fields H. W., Sarver D. M. (2006). *Contemporary Orthodontics: Elsevier Health Sciences*.

[12] Skaik A., Wei X. L., Abusamak I., Iddi I. (2019). Effects of time and clear aligner removal frequency on the force delivered by different polyethylene terephthalate glycol-modified materials determined with thin-film pressure sensors. *American Journal of Orthodontics and Dentofacial Orthopedics*.

[13] Vlaskalic V., Boyd R. (2002). Clinical evolution of the Invisalign appliance. *Journal of the California Dental Association*.

[14] Womack W. R., Day R. H. (2008). Surgical-orthodontic treatment using the Invisalign system. *Journal of Clinical Orthodontics*.

